# Metachronous bilateral spontaneous spermatic vein thrombosis: A rare cause of orchialgia

**DOI:** 10.1016/j.eucr.2022.102234

**Published:** 2022-09-16

**Authors:** Ibrahim A. Khalil, Hamed Mohammed, Maya Aldeeb, Mohamed Hatem, Ammar Alani, Khalid Al-Jalham

**Affiliations:** a Department of Urology, Hamad Medical Corporation, Doha, Qatar; b Department of Medical Education, Medical Intern, Hamad Medical Corporation, Doha, Qatar; c Department of Medical Education, Family Medicine Residency Program, Hamad Medical Corporation, Doha, Qatar

**Keywords:** Spermatic vein thrombosis, Orchialgia, Scrotal pain, Rare cause

## Abstract

Scrotal pain and swelling are common presentation, the prompt evaluation and diagnosis is needed due to wide range of causes, uncommon causes of orchialgia should be kept in mind whenever there is no clear diagnosis. Spermatic vein thrombosis usually presents with orchialgia along with episodes of acute exacerbation of pain. The diagnosis is challenging and need high index of suspension and detailed radiological evaluation. We present a case of metachronous bilateral unprovoked spermatic vein thrombosis treated conservatively with anticoagulation with good response and resolution of symptoms.

## Abbreviations

US: Ultrasound
TVT: Testicular venous thrombosis
NOA: New Oral Anticoagulants

## 1 Introduction

Scrotal pain and swelling is common presentation, the prompt evaluation and diagnosis is needed due to wide range of causes that might need urgent intervention as torsion to more chronic cases like varicocele.^1^ Nevertheless the differential diagnosis included uncommon cases for testicular pain like spermatic vein thrombosis that requires high suspicion with detailed evaluation and dictated radiological studies to start and guide the management. In this case report we are highlighting a rare presentation of bilateral testicular pain due to metachronous development of bilateral idiopathic spermatic vein thrombosis.

### 1.1 Case presentation

**Fig. 1:**
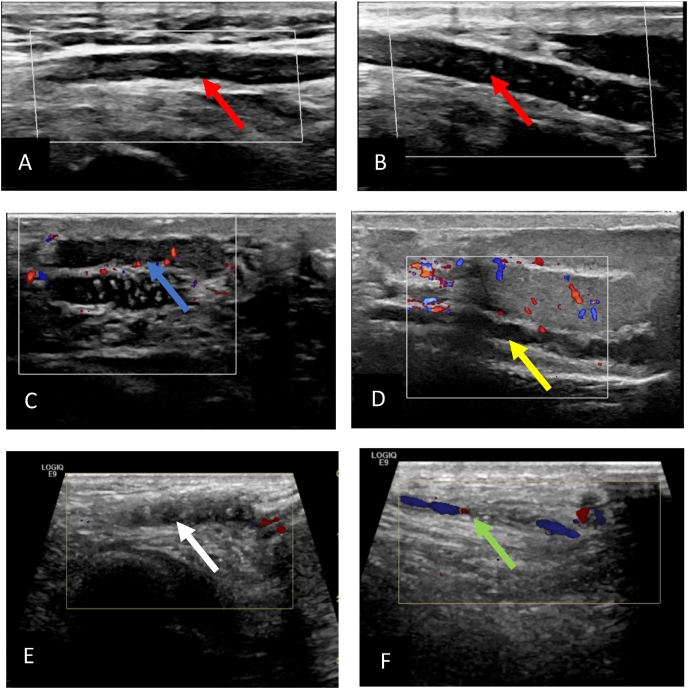
Doppler ultrasound showing A and B showing left spermatic vein thrombosis with flow on (Red arrow), C: left spermatic vein thrombosis on follow up images (blue arrow), D right spermatic vein thrombosis (yellow Arrow), E: perisistance of left pseramtoc vin thrombosis after 6 month of anticoagulation (white Arrow) while F showing resolution of the right spermatic vien thrombosis with flow on doppler images after antigoagulation (green arrow). (For interpretation of the references to colour in this figure legend, the reader is referred to the Web version of this article.)

A40-year-old healthy gentleman presented to emergency department initially with an exacerbation of left scrotal pain has been there for 3 months, on and off with no associated fever or other features of orchitis, no trauma or strenuous activity. On physical examination left testis was firm, tender without swelling. Inflammatory markers within normal range. A Scrotal Doppler ultrasound (US) showed a non-compressible thrombus extending from the left spermatic to the inguinal veins (Fig. 1 A and B) with normal testicular vascularity and patent right spermatic vein. Patient was treated conservatively with analgesia, scrotal support, and started on rivaroxiban 20 mg. Upon follow up after one month he was complaining of bilateral scrotal pain, US doppler showed persistence of left spermatic veins thrombosis (Fig. 1C) and development of new right spermatic vein thrombosis (Fig. 1D). Patient continued on rivaroxiban and in his follow up after 4 months patient symptoms improved and US doppler showed regression of the left spermatic vein thrombosis (Fig. 1E)and resolution of the right spermatic vein thrombosis (Fig. 1F).

Patient had work up to rule out coagulopathy patient had normal Coagulation profile, Negative lupus anticoagulant, *anti*-cardiolipin, and Factor V leiden mutation. The only risk factor for deep vein thrombosis was smoking which is a modifiable risk factor and patient quit smoking.

## 2 Discussion

Differential diagnosis of chronic testicular pain includes Varicocele, Testicular mass, Spermatocele, Hydrocele, post-procedural pain, and hernia.^1^ Testicular vein thrombosis (TVT) is an unusual pathology to keep in mind whenever there is no clear cause of testicular pain as it is easily misdiagnosed and confused with other pathologies because of the lack of specific clinical features and clear distinguishable symptoms as it might present with chronic or acute testicular pain^2^. Commonly, Thrombosis affects the left spermatic vein, and the patient usually complains of pain and swelling, and are the most frequent symptoms and these symptoms worsen over days to weeks. Clinically It is a very challenging diagnosis that requires evaluation using a Doppler Ultrasound scan of the scrotum and testis for an accurate diagnosis.^1^, ^2^, ^3^ The doppler ultrasound is needed as is will to prove that there is no flow in the obstructed spermatic vein which is which the presence of the incompressible thrombus is diagnostic of spermatic vein thrombosis.

The origin of testicular venous thrombosis remains unclear. Although a lot of physicians and researchers relate it to pathological changes like varicocele, obstruction of the venous drainage (renal tumors, trauma, heavy exercises, or prolonged sexual activity), coagulopathies, or other systemic diseases,^4^ on the other hand in his series Lenz et al. found an association between TVT with previous malignant pathologies in the abdominal cavity. He suggests that once spermatic vein thrombosis was diagnosed, the patient should be carefully assessed for underlying malignancy conditions.^3^ But in most of the cases there was no clear cause of this phenomena.

The nature history of TVT varies as there it might resole spontaneously or to be complicated with pulmonary embolism.^2^^,^^5^ The initial treatment of TVT is medical treatment with anti-inflammatory, Analgesia, and scrotal support.^5^ There Is still no clear consensus about the management, conservative with medical treatment or surgical, with few cases published in the literature, but as it is considered a deep vein thrombosis an anticoagulation is warranted from 3 to 6 months, with either warfarrin or new oral anticoagulants (NOAs) like rivaroxiban used in our case, the duration of anticoagulation depends on the risk of recurrence.^5^

We present a case of metachronous bilateral unprovoked spermatic vein thrombosis treated conservatively with NOAs with good response and resolution of symptoms.

## 3 Conclusion

Spermatic vein thrombosis is a rare diagnosis needs his clinical suspicion, doppler ultrasound is the preferred diagnostic modality to rule out other causes of testicular pain and confirm the diagnosis. The main stay of treatment is conservative with anticoagulation and analgesia.

## Declaration of competing interest

The authors declare that they have no financial or non-financial conflicts of interest related to the subject matter or materials discussed in the manuscript.
